# Substrain-specific behavioral variation in female C57BL/6 and C57BL/10 mice

**DOI:** 10.3389/fnbeh.2026.1805176

**Published:** 2026-04-15

**Authors:** Celine L. St. Pierre, Natalia M. Gonzales, Greta Sokoloff, Oksana Polesskaya

**Affiliations:** 1 Section of Genetic Medicine, University of Chicago, Chicago, IL, United States; 2 Department of Psychological and Brain Sciences, University of Iowa, Iowa City, Iowa, IA, United States; 3 Department of Psychiatry, University of California San Diego, La Jolla, CA, United States

**Keywords:** C57BL/10, C57BL/6, fear conditioning, forced swim test, locomotor response to cocaine, prepulse inhibition, sensorimotor gating

## Abstract

**Introduction:**

Inbred mouse strains are essential to biomedical research, yet accumulating mutations and substrain divergence introduce phenotypic variability that can confound experimental outcomes. This study investigates behavioral differences among 13 inbred mouse substrains: eight C57BL/6 (B6) and five C57BL/10 (B10), bred in-house to control for environmental effects.

**Methods:**

Female F1 offspring underwent a standardized battery of behavioral assays—open field test (OFT), locomotor response to cocaine (LOCO), fear conditioning (FC), prepulse inhibition (PPI), and the forced swim test (FST)—chosen for their relevance to models of psychiatric and substance use disorders.

**Results:**

Significant substrain-specific differences were observed across all behaviors. In the OFT, B6 substrains such as C57BL/6J showed higher activity than others, while B10 substrains exhibited distinct edge-zone preference patterns. Cocaine-induced locomotor stimulation varied significantly among B6 substrains but not among B10. In FC, substrain differences emerged in pre-training, contextual, and cued freezing behavior, particularly among B10 substrains. PPI testing revealed substrain-dependent variation in acoustic startle responses, with C57BL/10J displaying consistently lower startle amplitudes. In the FST, substrain-specific differences in swimming velocity and high mobility duration and frequency were found within the B6 group, while swimming distance showed substrain variation within the B10 group.

**Discussion:**

These findings demonstrate substantial phenotypic variability among closely related substrains, underscoring the critical importance of substrain selection in behavioral research. By focusing on female mice (a group underrepresented in prior work), this study addresses an essential gap and provides insights for researchers designing preclinical models of psychiatric disorders. The results provide the basis for studies in reduced complexity crosses to identify causal genetic variants underlying behavioral traits.

## Introduction

The development of inbred mouse strains from original fancy mice has been foundational in mouse genetics. The utility of these inbred strains comes from the assumption that all individuals within a strain are genetically identical. However, it's well-understood that new mutations continue to accumulate in inbred strains over time, leading to genetic and phenotypic variations among substrains. The C57BL lineage, established in the 1920s, has given rise to several substrains, notably C57BL/6 (B6) and C57BL/10 (B10) substrains, by 1937 [Festing, 1989; MGI (Mouse Genome Informatics), n.d.]. Since then, numerous B6 and B10 substrains have been developed and maintained across multiple facilities (reviewed in Mekada and Yoshiki, 2021). Although the substrains are genetically similar, they are not identical, and studies have found phenotypic and genetic differences that can influence experimental outcomes.

There are several reports that highlight differences between B6 and B10 substrains. Two B6 and B10 substrains, C57BL/6NCrlBR and C57BL/10J, were tested for fear memory and associated long-term potentiation (LTP) in the basolateral amygdala, hippocampal Schaeffer collateral-commissural and medial perforant pathway (Schimanski and Nguyen, 2005). The study reported that both B6 and B10 mice show normal fear memory and extinction, as well as normal synaptic physiology. Another study reported similar preferences for novelty or familiarity in C57BL/6 and C57BL/10 mice (substrain not specified) (Beery et al., 2018). (Deacon et al. 2007) compared C57BL/6JOlaHsd and C57BL/10ScSnOlaHsd female mice and found that C57BL/6 performed better than C57BL/10 in burrowing and digging test. The B6 and B10 strains differ in a number of physiological traits; B10 lifespan is longer, and the body size is larger, compared to B6 (MGI (Mouse Genome Informatics), n.d.). There are several immunological and oncological traits that are characteristic of B10. For example, C57BL/6J (B6) and C57BL/10SnJ (B10) differ in immune responses when infected with *Schistosoma mansoni*: B10 mice exhibit higher immunopathology and Th17-cell responses, increased production of pro-inflammatory cytokines, and a failure to attain alternative activation (Smith et al., 2015). B10 mice are prone to lymphoma (Smith et al., 1973) and are noted for immunological aberrations (MGI (Mouse Genome Informatics), n.d.).

In addition to the differences between B10 and B6 substrains, there are differences and variability within B6 and B10 substrains that were also examined in several studies. There is extensive evidence of behavioral differences between B6 substrains, such as differences in locomotor activity, learning, fear conditioning, nociception, and drug-related behaviors (Clapcote and Roder, 2004; Bothe et al., 2005; Grottick et al., 2005; Bryant et al., 2008; Mulligan et al., 2008; Matsuo et al., 2010; Ashworth et al., 2015; Mekada and Yoshiki, 2021). C57BL/6J and C57BL/6N substrains differ in several aspects of immune responses, as well as in microbiome composition (Hiergeist et al., 2025). For B10 substrains, the variability was shown for seizure thresholds (Grottick et al., 2005; Kadiyala et al., 2014). There is very little research that addresses behavioral differences among B10 substrains, in addition to (Denenberg, 1959) that reported differences in conditioning and response topography. Our work breaches this gap, providing a comprehensive comparison between five B10 substrains, as well as eight B6 substrains by testing them in parallel in a battery of behavioral tests.

From genetic point of view, the B10 and B6 strains differ at several genetic loci. It is well-documented that C57BL/6J has accumulated functionally significant mutations over time (Mulligan et al., 2008), and that various substrains of B6 segregate functionally important mutations (Kumar et al., 2013), including copy number variations (Egan et al., 2007). A recent study reported whole-genome comparison of eight B6 and five B10 substrains and identified hundreds of thousands of genetic differences (Mortazavi et al., 2022). That work showed that B6 and B10 lineages differ by more than 300,000 single nucleotide polymorphisms (SNPs) that were clustered into 28 short segments that are likely a result of introgressed haplotypes rather than new mutations. These genetic differences included protein-truncating variants, frameshifting indels and variants associated with differential expression (Mortazavi et al., 2022).

This study uses a panel of eight B6 and five B10 mouse substrains obtained from four commercial vendors. The substrains were selected based on their frequent use in biomedical research. These are the same substrains that were used for genomic analysis in Mortazavi et al. (2022). The purpose of this study is to further explore the degree of behavioral differences that can be expected among B6 substrains as well as to characterize the behavioral profiles of B10 substrains. The mice were tested in a series of behavioral tests to assess phenotypes relevant to substance use and psychiatric disorders. The tests included open field test (OFT) to assess behavioral responses to a novel environment, locomotor response to cocaine (LOCO) to evaluate locomotor response to cocaine administration, fear conditioning (FC) to measure response to fear stimulus, prepulse inhibition (PPI) to assess sensorimotor gating, and forced swim test (FST) to measure depression-like behaviors. We report significant substrain-specific differences that were observed across all behaviors, underscoring the importance of considering substrain-specific differences in experimental design and interpretation.

## Methods

### Mouse husbandry

**Figure 1:**
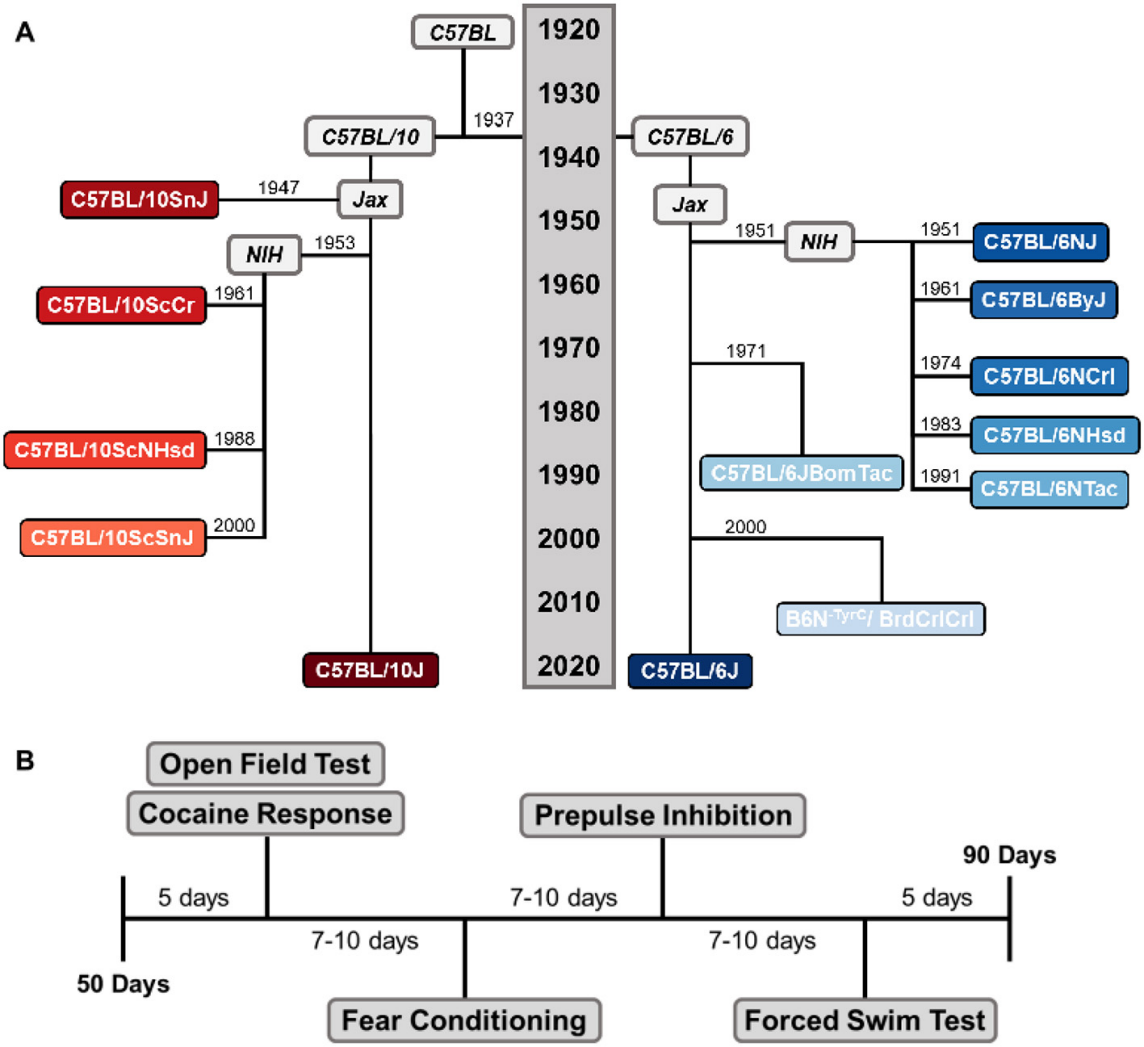
Chronological tree and experimental design of the C57BL substrains. **(A)** The chronological tree of the thirteen C57BL/6 and C57BL/10 substrains since C.C. Little's initial C57BL colony in 1921. Major events that established each substrain are positioned along the y-axis. Today, the B6 and B10 substrains are separated by over 300 generations. B10 substrains are shown in red **(left)** and B6 substrains are shown in blue **(right)**. Position along the x-axis does not convey any measure of time or relatedness. **(B)** Timeline of the behavioral testing battery. Mice completed the open field, locomotor response to 10 mg/kg of cocaine, conditioned fear, prepulse inhibition, and forced swim tests. Mice rested for a week in between each test.

We selected a panel of eight C57BL/6 (B6) and five C57BL/10 (B10) substrains that are often used in biomedical research, from four commercial vendors: C57BL/6J, C57BL/6NJ, C57BL/6ByJ, C57BL/10J, C57BL/10SnJ, C57BL/10ScSnJ, and C57BL/10ScCr mice from The Jackson Laboratory (Bar Harbor, ME, USA), C57BL/6JBomTac and C57BL/6NTac mice from Taconic Biosciences, Inc. (Germantown, NY, USA), C57BL/6NCrl and B6N^−*Tyrc*−*Brd*^/BrdCrCrl mice from Charles River Laboratories, Inc. (Wilmington, MA, USA), and C57BL/6NHsd and C57BL/10ScNHsd mice from Harlan Sprague Dawley, Inc. (now Envigo; Indianapolis, IN, USA). The historical relationships of these B6 and B10 substrains over the past 100 years are shown in Figure 1A.

Males and females from each substrain were obtained from their vendors at 6–8 weeks old, acclimated for 1 week, and placed in breeding harems. We bred F1 offspring for each substrain in our colony to eliminate any effects of shipping stress or different vendor environments on behavioral phenotypes. Females were single-housed once visibly pregnant. F1 offspring were weaned at 21–28 days and housed 4–5 in single-sex cages. Mice were fed *ad libitum* on standard lab chow, received 2 × 2 inch cotton nestlets as environmental enrichment, maintained on a 12-h light/dark cycle, and phenotyped during the light phase. The estrous cycle was not examined. After testing, mice were euthanized by CO_2_ asphyxiation followed by cervical dislocation. All experiments were approved by the Institutional Animal Care and Use Committee at the University of Chicago (Chicago, IL, USA) in accordance with the National Institutes of Health guidelines.

### Behavioral test battery

We subjected the female F1 offspring to a 4-week battery of the following behavioral tests: the open field (OFT), locomotor response to 10 mg/kg cocaine (LOCO), conditioned fear (FC), prepulse inhibition (PPI), and forced swim tests (FST; Figure 1B). We tested 9–14 mice per substrain (median = 12, total = 152) in six cohorts of 11–42 individuals. Their median age was 54 days at the start of testing (range = 50–69) and 102 days at death (range = 95–114). B6N^−*Tyrc*−*Brd*^/BrdCrCrl mice completed the same testing battery as the other substrains, but they were removed from analyses for conditioned fear and forced swim tests because their white coat colors interfered with the cameras' ability to track movement. They were included in the remaining analyses because those tests use infrared photobeams or piezoelectric sensors to detect movement in a manner impervious to coat color.

### Open field test (OFT) and locomotor response to cocaine (LOCO)

We measured locomotor activity using automated VersaMax activity chambers (AccuScan Instruments Inc., Columbus, OH, USA) as previously described (Bryant et al., 2009). Briefly, chambers consisted of a clear acrylic arena (42 × 42 × 30 cm) held within a frame of evenly spaced photocells and receptors, resulting in a dense two-dimensional grid of infrared photobeams. Beam breaks were recorded and converted into distance traveled (cm). Each activity chamber was dimly lit (~80 lux) and housed inside a sound-attenuating PVC/Lexan environmental chamber (Med Associates Inc., Georgia, VT, USA). Fans masked background noise and provided ventilation for each chamber.

We used 152 female mice (9–13 mice per strain) that were 7–10 weeks old (Figure 1B, Table 1). We tested mice over three consecutive days. Each day, mice were transported from the vivarium to the testing room, habituated in their home cages for 30 min, weighed, and then placed into individual holding cages filled with clean bedding. Mice received an intraperitoneal injection of physiological saline (first and second days) or 10 mg/kg cocaine (third day). All systemic injections were administered in a volume of 10 mL/kg body weight. Afterwards, mice were immediately placed into individual activity chambers. Their locomotor activity was recorded over 15 min, but we only analyzed the first 5-min interval. This interval was selected because cocaine-induced hyperlocomotion peaks rapidly following intraperitoneal injection and then declines, and because novelty-induced locomotion habituates quickly, making the first 5-min bin the most sensitive window for detecting substrain differences (Bryant et al., 2008, 2009). We measured distance traveled including activity in the defined “center” (a square 8.3 cm from each wall) and “edge” zones.

**Table 1:** Sample size for behavioral tests.

Substrain	OFT	LOCO	FC	PPI	FST
C57BL/6J	12	12	12	12	12
C57BL/6NJ	11	11	11	11	11
C57BL/6ByJ	12	12	12	12	12
C57BL/6NCrl	9	9	9	9	9
C57BL/6NTac	12	12	12	12	12
C57BL/6NHsd	14	14	14	14	14
C57BL/6JBomTac	12	12	12	12	12
B6N^−*Tyrc*−*Brd*^/BrdCrCrl	10	10	–	10	–
C57BL/10J	12	12	12	12	12
C57BL/10SnJ	13	13	13	13	13
C57BL/10ScSnJ	12	12	12	12	12
C57BL/10ScCr	10	10	10	10	10
C57BL/10ScNHsd	13	13	13	13	13

### Fear conditioning (FC)

We estimated freezing behaviors using the ActiMetrics FreezeFrame system (Wilmette, IL, USA), as previously described (Ponder et al., 2007; Bryant et al., 2008). Briefly, we tested mice over three consecutive days. Each day, mice were transported from the vivarium to the testing room, habituated in their home cages for 30 min, and placed into individual holding cages with clean bedding. Mice were then placed into the conditioning chamber for a 5 min training session, described below. We excluded the initial 30 s of each training session due to high variability in freezing behavior during that period.

We used 9–13 female mice per substrain, at 9 weeks of age (Figure 1B, Table 1). We measured all variables as the percentage of time spent freezing during the indicated time interval. On day 1, we defined “pre-training freezing” as the period before the paired stimuli (30–180 s). After 3 min, mice received two trials of a 30 s, 3 kHz, 85–90 dB tone (conditioned stimulus, CS) that co-terminated with a 2-s, 0.5 mA foot shock (unconditioned stimulus, US). Trials were separated by a 30-s interval. We defined “freezing to tone (day 1)” as the average of these two tone presentations (180–210 s and 240–270 s). On day 2, mice were placed into the same testing chamber as day 1, but no tones (CS) or shocks (US) were presented. We defined “freezing to context” as the same period as the pre-training phase (30–180 s). On day 3, mice were placed into contextually-altered chambers with different olfactory, visual, tactile, and auditory stimuli. We defined “freezing to an altered context” as the same pre-training period (30–180 s). After 3 min, mice received two more trials of auditory tones (CS) in the same manner as day 1, however, no foot shocks (US) were delivered. We defined “freezing to tone (day 3)” as the average of these two tone presentations (180–210 s and 240–270 s).

### Prepulse inhibition (PPI)

We measured prepulse inhibition of the startle response using the SR-Lab Startle Response system (San Diego Instruments, San Diego, CA, USA), as previously reported (Palmer and Airey, 2003; Samocha et al., 2010). Briefly, mice were transported from the vivarium to a holding room and habituated in their home cages for 30 min. Immediately before testing, mice were placed into individual holding cages with clean bedding and moved into the adjacent testing room. Each mouse was placed into a cylindrical Plexiglass container (5 cm in diameter) on a platform contained within an illuminated, ventilated testing chamber. The cylinder was connected to a piezoelectric accelerometer that measured the mouse's movements. We calibrated the accelerometer each day before testing began.

Each chamber had 70 dB of white background noise that persisted throughout the session. The testing protocol began with a 5 min acclimation to the background noise followed by 62 trials of one of the following five types: a “no stimulus trial” (no acoustic stimulus presented), a “pulse-alone trial” (40 ms, 120 dB acoustic burst), and three “prepulse trials” (20 ms prepulse that was 3, 6, or 12 dB above the 70 dB background noise followed 100 ms later by a 40 ms, 120 dB pulse). Trials were arranged into four consecutive blocks. Blocks 1 and 4 were pulse-alone trials. Blocks 2 and 3 contained a pseudorandom mixture of pulse-alone, no stimulus, and each prepulse trial (3, 6, and 12 dB). Startle responses were recorded for 65 ms after the start of the 120 dB stimulus. The intertrial interval was 9–20s (mean = 15 s) throughout the test.

We quantified PPI at each intensity (3, 6, or 12 dB) as the difference between mean startle amplitudes (SA) during the prepulse trials and pulse-alone trials, normalized by the pulse-alone trials:


$$\begin{array}{l} \text{PPI}=\left(\text{SApulse SAprepulse}\right)\;/\text{ SApulse} \end{array}$$ (1)

We defined “average PPI” as the mean startle inhibition across all three prepulse intensities and “startle” as the mean startle amplitude across all pulse-alone trials (no prepulse). The “no stimulus” trials were used to identify technical problems and were not used to calculate any phenotypes.

### Forced swim test (FST)

We measured depression-like behaviors using the Noldus Ethovision XT system (Noldus Information Technology, Lessburg, VA, USA), as previously reported (Sittig et al., 2016). Briefly, mice were transported from the vivarium to the testing room, habituated in their home cages for 30 min, and placed into individual holding cages with clean bedding. Each mouse was placed into a white polyethylene bucket (23.5 cm depth × 15.5 cm diameter) filled with warm water (23–25 °C; 11.7 cm deep) for 6 min (Porsolt et al., 1977; Lucki et al., 2001). Sessions were digitally recorded with a tripod-mounted video camera positioned directly above the test buckets.

The last 4 min of their swimming activity was scored by the Ethovision XT v5.1 software. We quantified the total distance (cm) and mean velocity (cm/s) of swimming around the test bucket. We differentiated between vigorous activity (“highly mobile”) and floating (“immobile”). We defined “duration of high mobility” as the number of seconds spent with >10% movement between frames.

### Statistical analysis

To focus on substrain differences within each branch, we analyzed B6 and B10 data separately. Behavioral data was quantile-normalized by phenotype and checked for outliers, where outliers were defined as ± 3 standard deviations. No outliers were detected. We fit a series of one-way ANOVA models to screen for potential covariates affecting the phenotypes: age at testing, testing chamber, time of day, testing trial/run, body weight at testing (OFT/LOCO), and the number of fecal boli (OFT/LOCO). We identified the testing chamber as a significant covariate for each behavioral test (*p* ≤ 0.05), so we extracted the phenotype residuals from those ANOVAs to remove any phenotypic variation due to the testing chamber. Next, we used another series of ANOVA models to test whether the residualized phenotypes were influenced by substrain. We considered FDR-corrected *p*-values ≤ 0.1 to be significant. For significant substrain effects, we calculated eta-squared (η^2^) as a measure of effect size, representing the proportion of total phenotypic variance explained by substrain. These results are reported in Table 2. For phenotypes with significant strain effects, we conducted Fisher's Least Significant Differences *post-hoc* tests to identify substrains with significant behavioral differences (adjusted *p* ≤ 0.05) These results are reported in Supplementary Table 1. Finally, we also directly compared the canonical JAX substrains of each branch (C57BL/6J and C57BL/10J) with one-way ANOVAs to assess significant behavioral differences (*p* ≤ 0.05) between these two commonly used strains (Table 3). To quantify the effect size of behavioral differences between the two canonical substrains, C57BL/6J and C57BL/10J, we calculated Cohen's d as the difference between group means divided by the pooled standard deviation.

**Table 2:** ANOVA for substrain-by-phenotype comparisons.

Branch	Phenotype	*F*_stat	*p*_value	p_fdr	η^2^	Significant
Open field test						
B6	Total distance traveled	6.3937	0.0000	0.0001	0.348	True
B6	Percentage of movement within the edge zone of the arena	0.6066	0.7489	0.7748	0.048	False
B10	Total distance traveled	2.5715	0.0478	0.0956	0.158	True
B10	Percentage of movement within the edge zone of the arena	2.9278	0.0288	0.0666	0.176	True
Locomotor response to cocaine						
B6	Change in total distance traveled	1.9531	0.0712	0.0929	0.14	True
B6	Change in novelty ratio	2.0579	0.0571	0.0779	0.146	True
B10	Change in total distance traveled	0.3673	0.8309	0.8902	0.026	False
B10	Change in novelty ratio	1.1352	0.3496	0.4994	0.076	False
Fear conditioning						
B6	Pre-training baseline	3.2663	0.0066	0.0254	0.207	True
B6	Freezing to the training context	1.1625	0.3355	0.3594	0.085	False
B6	Freezing to the altered context	2.2528	0.0472	0.0705	0.153	True
B6	Freezing to tone	1.8837	0.0947	0.1183	0.131	False
B10	Pre-training baseline	2.1521	0.0866	0.1528	0.135	False
B10	Freezing to the training context	13.4585	0.0000	0.0000	0.495	True
B10	Freezing to the altered context	0.9660	0.4336	0.5655	0.066	False
B10	Freezing to tone	6.7049	0.0002	0.0018	0.328	True
Prepulse inhibition						
B6	PPI at prepulse intensity of 3 dB	4.6411	0.0002	0.0015	0.281	True
B6	PPI at prepulse intensity of 6 dB	3.8288	0.0012	0.0059	0.244	True
B6	PPI at prepulse intensity of 12 dB	2.1283	0.0493	0.0705	0.152	True
B6	Startle amplitude	2.2647	0.0368	0.0705	0.16	True
B10	PPI at prepulse intensity of 3 dB	3.2040	0.0195	0.0627	0.189	True
B10	PPI at prepulse intensity of 6 dB	4.2930	0.0043	0.0214	0.238	True
B10	PPI at prepulse intensity of 12 dB	7.9706	0.0000	0.0006	0.367	True
B10	Startle amplitude	5.2711	0.0011	0.0086	0.277	True
Forced swim test						
B6	Total distance swum	1.6046	0.0675	0.0900	0.114	False
B6	Mean swimming velocity	1.9905	0.1577	0.1819	0.114	False
B6	Duration of highly mobile swimming	2.6245	0.0230	0.0590	0.174	True
B10	Total distance swum	3.9070	0.0073	0.0274	0.221	True
B10	Mean swimming velocity	0.2311	0.9198	0.9198	0.221	False
B10	Duration of highly mobile swimming	2.2664	0.0737	0.1381	0.142	False

**Table 3:** Comparison between commonly used strains C57BL/6J and C57BL/10J.

Test	Phenotype	*F*_stat	*p*_value	Cohen's *d*	Significant
OFT	Total distance traveled	2.3970	0.1358	0.632	False
OFT	Percentage of movement within the edge zone of the arena	5.0860	0.0344	0.921	True
LOCO	Total distance traveled	4.4489	0.0465	0.861	True
LOCO	Percentage of movement within the edge zone of the arena	0.0229	0.8811	0.062	False
FC	Pre-training baseline	0.5816	0.4538	−0.311	False
FC	Freezing to the training context	12.4950	0.0019	−1.443	True
FC	Freezing to the altered context	2.6436	0.1182	−0.664	False
FC	Freezing to tone	16.8619	0.0005	−1.676	True
PPI	PPI at prepulse intensity of 3 dB	14.8180	0.0009	1.572	True
PPI	PPI at prepulse intensity of 6 dB	21.5379	0.0001	1.895	True
PPI	PPI at prepulse intensity of 12 dB	10.2269	0.0042	1.306	True
PPI	Startle amplitude	2.9372	0.1006	−0.7	False
FST	Total distance swum	1.7166	0.2036	0.535	False
FST	Mean swimming velocity	1.7166	0.2036	0.535	False
FST	Duration of highly mobile swimming	1.5921	0.2202	0.515	False

We conducted all analyses in R using various packages for data manipulation, *post-hoc* comparisons, and data visualization.

## Results

We used a panel of eight C57BL/6 (B6) and five C57BL/10 (B10) mouse substrains to assess behavioral responses of female mice in a battery of behavioral tests relevant to substance use and psychiatric disorders, in order to characterize differences between B6 and B10 groups, and substrain-specific differences. We bred the mice in-house and used F1 offspring to minimize environmental differences related to the breeding facility and shipping. The tests included open field test (OFT) to assess behavioral responses to a novel environment, locomotor response to cocaine (LOCO), fear conditioning (FC) to measure response to fear, prepulse inhibition (PPI) to assess sensorimotor gating, and forced swim test (FST) to measure depression-like behaviors.

### Open field test (OFT)

Locomotor activity was measured to evaluate baseline exploration and novelty-seeking behavior, as anxiety-related phenotypes, and to obtain background metrics for drug-induced hyperlocomotion.

**Figure 2:**
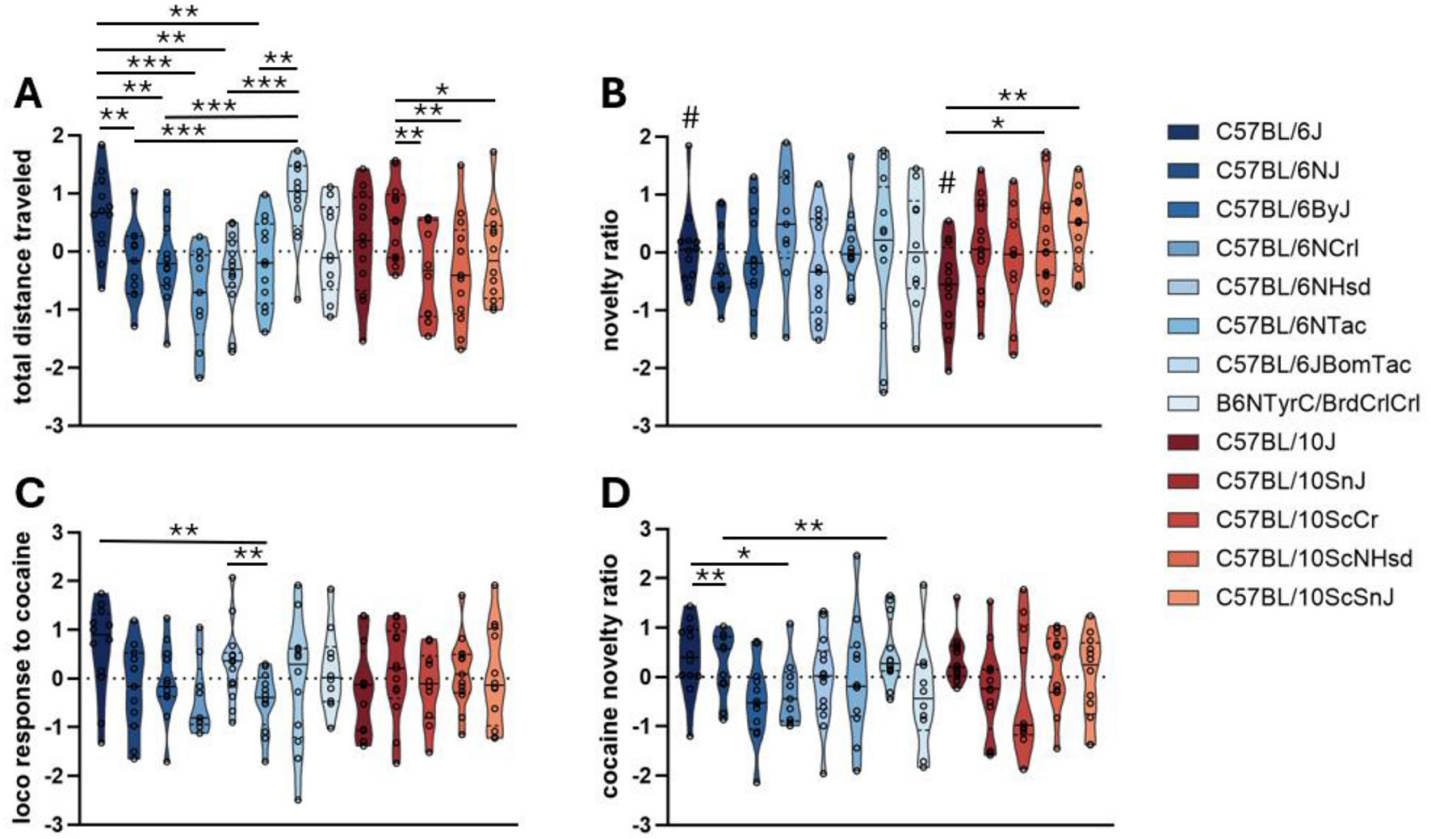
Open field and locomotor response to cocaine tests. The plots show truncated violin plots of residualized values. **(A)** Total distance traveled on day 1. **(B)** Novelty ratio on day 1 (the percentage of distance traveled in the defined “edge” zone). **(C)** Locomotor response to 10 mg/kg cocaine (change in distance traveled between day 3 (cocaine injection) and day 2 (saline injection) of testing). **(D)** Change in novelty ratio after 10 mg/kg cocaine (change in the percentage of distance traveled in the “edge” zone between day 3 (cocaine injection) and day 2 (saline injection) of testing. Substrains are ordered along the x-axis according to their time of separation from the JAX colony strains (C57BL/6J and C57BL/10J). C57BL/10 substrains are in shades of red; C57BL/6 substrains are in shades of blue. *p-value < 0.05, **p-value < 0.01, ***p-value < 0.001, assessed by ANOVA with Fisher's Least Significant Difference *post hoc* correction. # indicates that commonly used C57BL/6J and C57BL/10J strains significantly differ (*p*-value < 0.05). Bars represent the median value per substrain.

Significant effects of substrain for both B6 and B10 branches were observed for total distance traveled (Figure 2A). Among B6 substrains, C57BL/6J and C57BL/6JBomTac mice exhibited the highest levels of spontaneous locomotor activity, traveling significantly farther than B6 substrains C57BL/6NJ, C57BL/6ByJ, C57BL/6NCrl, C57BL/6NHsd, C57BL/6NTac, and B6N^−*Tyrc*−*Brd*^/BrdCrCrl. C57BL/6NCrl mice traveled significantly less distance than C57BL/6NJ and C57BL/6ByJ mice. Among B10 substrains, C57BL/10SnJ traveled significantly farther than C57BL/10ScCr and C57BL/10ScNHsd. There was no significant difference in distance traveled between the commonly used strains C57BL/6J and C57BL/10J.

Analysis of the novelty ratio, defined as the percentage of movement within the edge zone of the arena, revealed significant substrain effects within the B10 lineage but not within B6 lineage (Figure 2B). C57BL/10J mice spent less time in the edge zone compared to all other B10 substrains, as well as compared to C57BL/6J, indicating a reduction in anxiety-like behavior or increased willingness to explore the center of the arena.

ANOVA results for all substrain-by-phenotype comparisons are reported in Table 2. *Post-hoc* results are reported in Supplementary Table 1.

### Locomotor response to cocaine

To assess how acute cocaine exposure modulates locomotor activity and exploratory behavior, we measured the change in total distance traveled and the change in novelty ratio between days of saline and cocaine (10 mg/kg) injection.

The locomotor response to cocaine, defined as the change in total distance traveled between day 3 (cocaine injection) and day 2 (saline injection), revealed substrain variability (Figure 2C). A significant effect of substrain was observed for the B6 lineage, with C57BL/6J showing a greater locomotor response to cocaine than C57BL/6NCrl, C57BL/6NJ, and C57BL/6NTac; C57BL/6NHsd also showed a greater response than C57BL/6NTac. There was no significant effect of substrain for the B10 lineage. However, C57BL/6J mice had a significantly greater locomotor response than C57BL/10J mice, the two commonly used substrains.

The effect of cocaine on anxiety-related behavior, measured as the change in novelty ratio (percentage of movement within the edge zone), also varied by substrain (Figure 2D). A significant substrain effect was observed for the B6 lineage. Specifically, C57BL/6ByJ and B6N^−*Tyrc*−*Brd*^/BrdCrCrl mice spent less time in the edge zone after cocaine compared to C57BL/6J and C57BL/6JBomTac mice; C57BL/6JBomTac mice spent more time in the edge zone than C57BL/6NCrl mice. There was no significant effect of substrain for the B10 lineage. Notably, there was no significant difference in novelty ratio change between the commonly used C57BL/6J and C57BL/10J mice.

ANOVA results for all substrain-by-phenotype comparisons are reported in Table 2. *Post-hoc* results are reported in Supplementary Table 1.

### Fear conditioning

The FC test was used to evaluate both contextual memory (association with the training environment) and cued memory (association with an auditory tone) in response to aversive stimuli. This test models how animals learn to associate neutral cues or environments with aversive experiences. Freezing behavior, a mouse response to fear, was measured as an indicator of learned fear.

**Figure 3:**
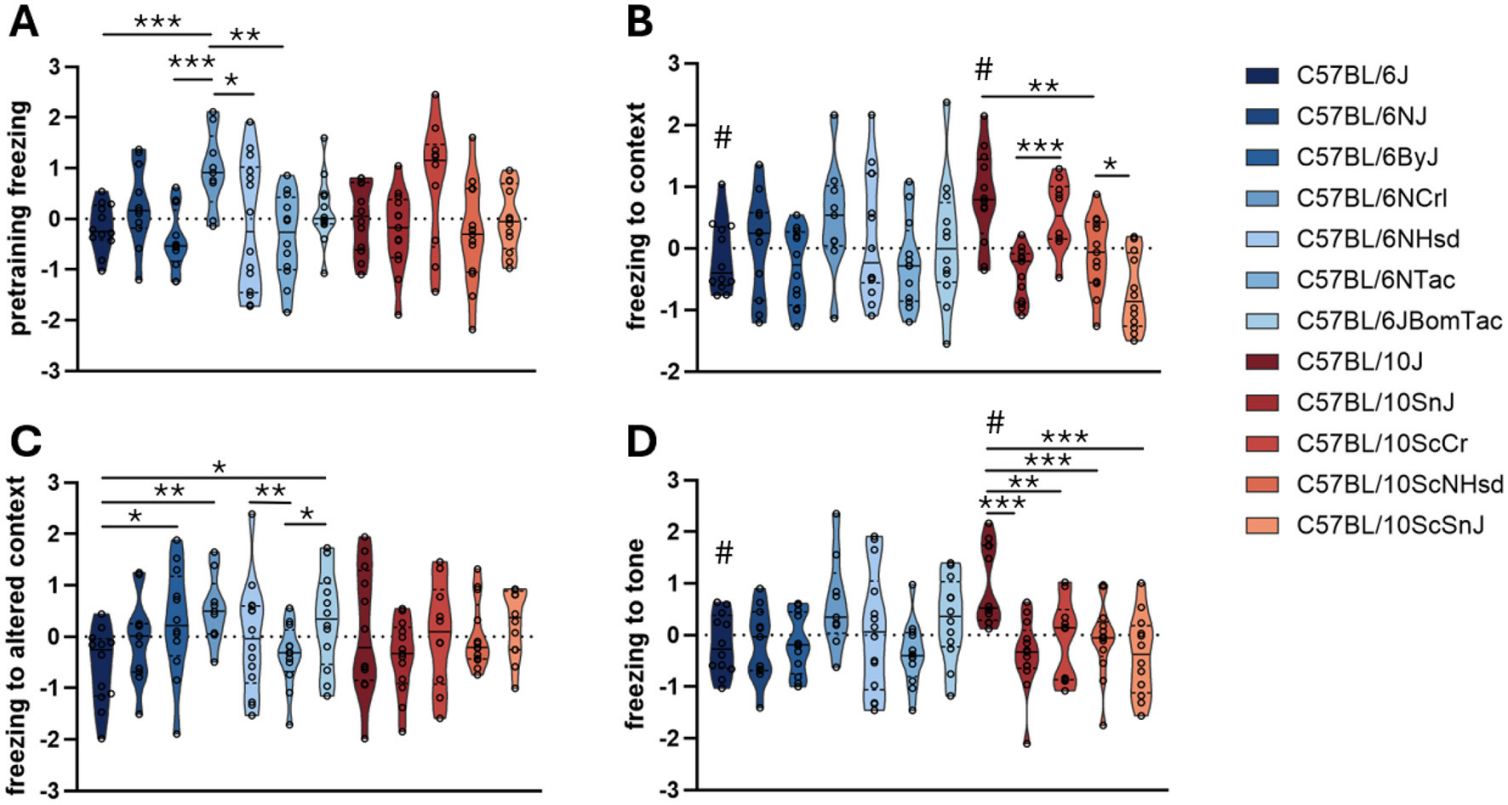
Fear Conditioning test. The plots show truncated violin plots of residualized values. **(A)** Average pre-training freezing (day 1). **(B)** Average freezing to context (day 2). **(C)** Average freezing to altered context (day 3). **(D)** Average conditioned freezing to tone (day 3). Substrains are ordered along the x-axis according to their time of separation from the JAX colony strains (C57BL/6J and C57BL/10J). C57BL/10 substrains are in shades of red; C57BL/6 substrains are in shades of blue. *p-value < 0.05, **p-value < 0.01, ***p-value < 0.001, assessed by ANOVA with Fisher's Least Significant Difference *post hoc* correction. # indicates that commonly used C57BL/6J and C57BL/10J strains significantly differ (*p*-value < 0.05). Bars represent median phenotypes per substrain.

Variability in pre-training baseline (the amount of time the mouse spends freezing in the new environment) demonstrates substrain variability in innate anxiety in a novel context (Figure 3A). B6 substrains showed variability, with C57BL/6NCrl mice exhibiting higher baseline freezing than C57BL/6J, C57BL/6ByJ and C57BL/6JBomTac. C57BL/6NCrl also froze more than C57BL/6NHsd and C57BL/6NTac. The overall effect for the B10 lineage was not statistically significant. C57BL/6J and C57BL/10J strains did not differ significantly for the pre-training baseline (Table 3).

Freezing to the training context was assessed on day 2 (Figure 3B), where higher levels of freezing indicate stronger contextual fear memory. B6 substrains showed little variability in contextual freezing, except for C57BL/6NCrl, which froze more than C57BL/6ByJ. B10 substrains demonstrated pronounced differences among substrains. C57BL/10J and C57BL/10ScCr mice demonstrated significantly greater contextual freezing than other B10 substrains. C57BL/10J mice froze more than C57BL/6J (Table 3), indicating a lineage-specific enhancement of contextual fear memory.

Freezing in an altered context on day 3 (Figure 3C) measured contextual discrimination and cognitive flexibility. A significant substrain effect was seen among B6 mice, with C57BL/6J freezing less than C57BL/6ByJ, C57BL/6JBomTac, and C57BL/6NCrl; C57BL/6NTac also froze less than C57BL/6NCrl. There was no significant effect of substrain among B10 mice. C57BL/6J and C57BL/10J strain behaviors did not significantly differ (Table 3).

Cued fear memory was measured as freezing to tone on day 3 (Figure 3D). A significant substrain effect was seen among B6 mice, with C57BL/6J freezing less than C57BL/6ByJ, C57BL/6JBomTac, and C57BL/6NCrl; C57BL/6NTac also froze less than C57BL/6NCrl. No significant differences were found among B10 substrains. C57BL/6J and C57BL/10J strain behaviors did not significantly differ (Table 3).

ANOVA results for all substrain-by-phenotype comparisons are reported in Table 2. *Post-hoc* results are reported in Supplementary Table 1.

### Prepulse inhibition

The startle response is an involuntary contraction of muscles caused by an intense, sudden onset stimulus. When a startling stimulus is preceded by a smaller “prepulse” stimulus, the startle response is inhibited; this process is known as prepulse inhibition (PPI). PPI is used as a measure of sensorimotor gating (Geyer and Swerdlow, 2001; Geyer et al., 2001), which is a process by which extraneous stimuli are filtered out to prevent cognitive overload (Mcghie and Chapman, 1961).

**Figure 4:**
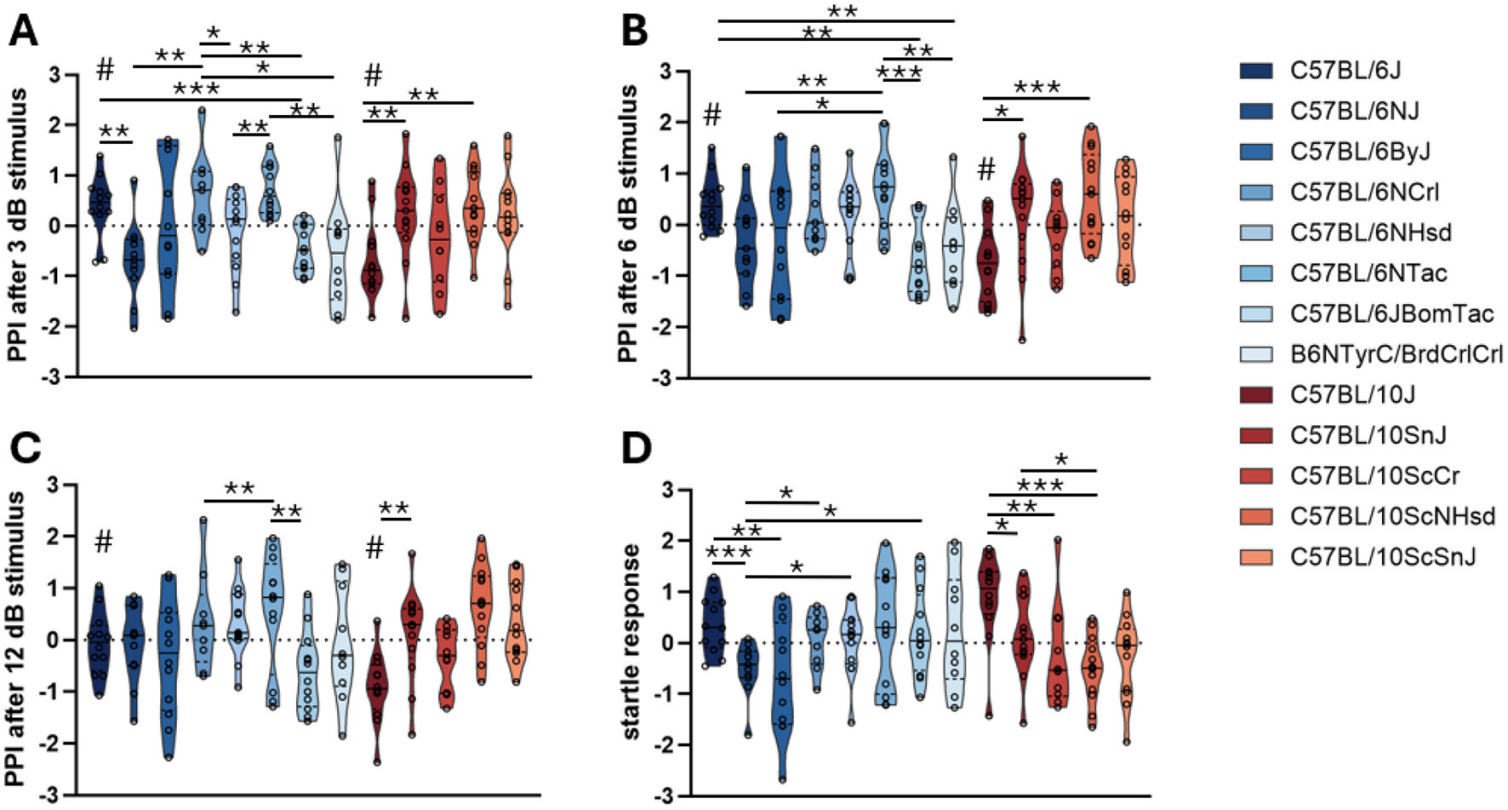
Prepulse inhibition test. The plots show truncated violin plots of residualized values. **(A)** PPI in response to 3 dB acoustic stimulus. **(B)** PPI in response to 6 dB acoustic stimulus. **(C)** PPI in response to 12 dB acoustic stimulus. **(D)** Startle amplitude in response to an acoustic stimulus. Substrains are ordered along the x-axis according to their time of separation from the JAX colony strains (C57BL/6J and C57BL/10J). C57BL/10 substrains are in shades of red; C57BL/6 substrains are in shades of blue. *p-value < 0.05, **p-value < 0.01, ***p-value < 0.001, assessed by ANOVA with Fisher's Least Significant Difference *post hoc* correction. # indicates significant difference between commonly used strains C57BL/6J and C57BL/10J (*p*-value < 0.05). Bars represent median phenotypes per substrain.

Across both B6 and B10 substrains, significant differences were observed in prepulse inhibition at multiple prepulse intensities. At the lowest prepulse intensity of 3 dB both lineages displayed robust substrain effects (Figure 4A). Among B6 mice, C57BL/6J, C57BL/6NCrl, and C57BL/6NTac exhibited significantly higher PPI compared to C57BL/6NJ and C57BL/6JBomTac; B6N^−*Tyrc*−*Brd*^/BrdCrCrl showed lower PPI than C57BL/6J, C57BL/6NCrl, and C57BL/6NTac. Among the B10 mice, C57BL/10J showed lower PPI than C57BL/10SnJ, C57BL/10ScNHsd, and C57BL/10ScSnJ. Comparing the two commonly used strains, C57BL/6J exhibited significantly higher PPI than C57BL/10J (Table 3).

These patterns persisted as the prepulse intensity increased to 6 dB (Figure 4B) and 12 dB (Figure 4C). C57BL/6NTac continued to demonstrate higher PPI than C57BL/6ByJ, C57BL/6JBomTac, and C57BL/6NJ, while C57BL/6JBomTac showed consistently lower PPI than C57BL/6NCrl and C57BL/6NHsd. Among B10 mice, C57BL/10J remained hypo-responsive compared to other B10 substrains, and C57BL/10ScCr also showed lower PPI than C57BL/10ScNHsd at 6 and 12 dB. C57BL/6J maintained significantly higher PPI than C57BL/10J across all prepulse intensities (Table 3).

When PPI was averaged across all prepulse intensities, the substrain differences remained. Among B6 mice, C57BL/6NTac and C57BL/6NCrl exhibited the highest average PPI, while C57BL/6JBomTac and C57BL/6NJ showed the lowest. Among B10 mice, C57BL/10J showed the lowest PPI, while C57BL/10ScCr also exhibited lower PPI than C57BL/10ScNHsd. C57BL/6J showed significantly higher average PPI than C57BL/10J.

Startle amplitude (measured during pulse-alone trials) also varied by substrain (Figure 4D). Among B6 mice, C57BL/6ByJ exhibited lower startle than C57BL/6J, C57BL/6JBomTac, C57BL/6NHsd, and C57BL/6NTac. Among B10 mice, C57BL/10J displayed the highest startle amplitude, significantly greater than all other B10 substrains. The difference in startle amplitude between C57BL/6J and C57BL/10J was not statistically significant (Table 3).

ANOVA results for all substrain-by-phenotype comparisons are reported in Table 2. *Post-hoc* results are reported in Supplementary Table 1.

### Forced swim test

The FST is a behavioral assay to measure depressive-like behaviors and active coping in rodents (Porsolt et al., 1977).

**Figure 5:**
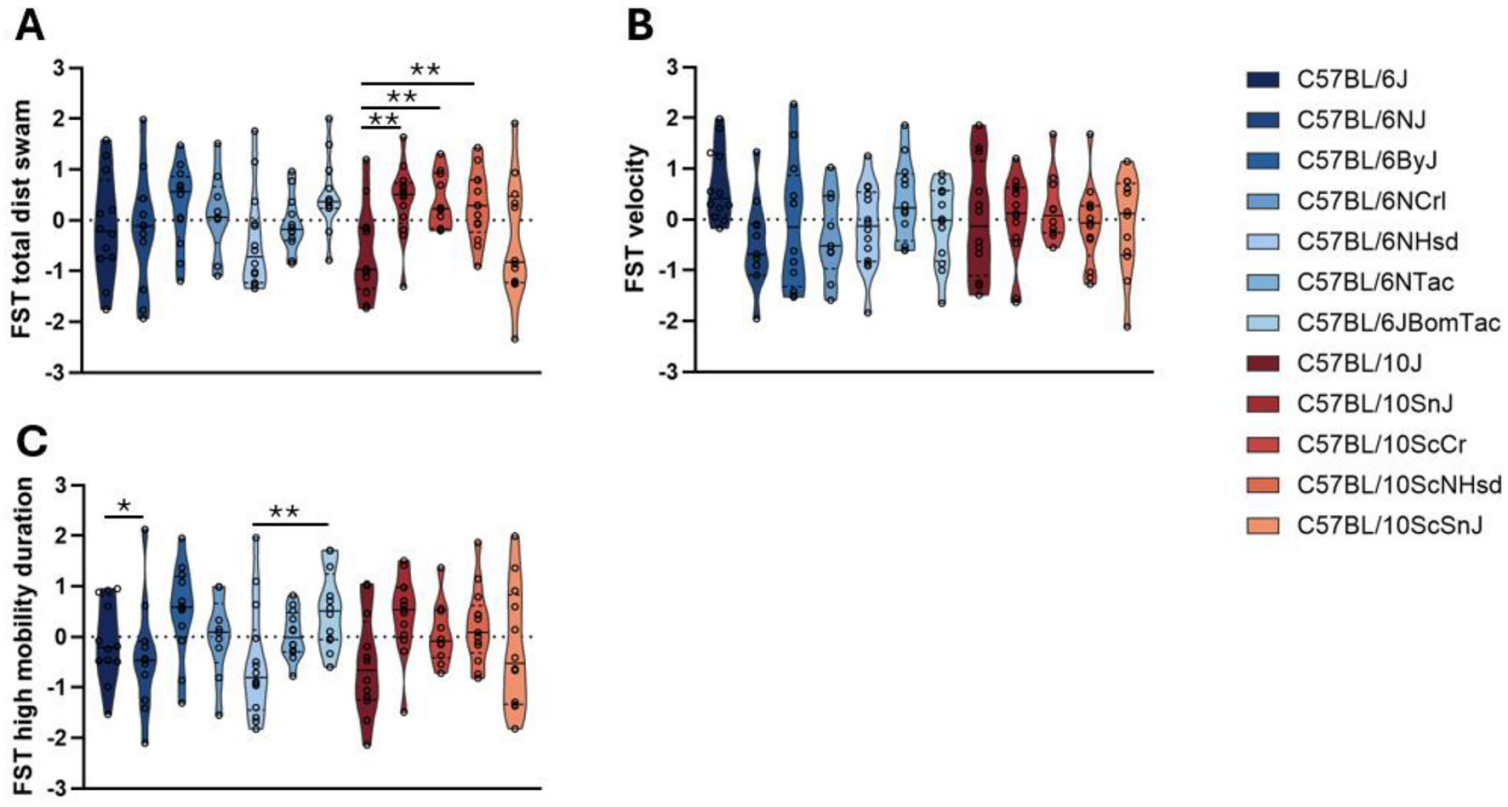
Forced swim test. **(A**) Total distance swum. **(B)** Average velocity. **(C)** Duration of high mobility swimming. Substrains are ordered along the x-axis according to their time of separation from the JAX colony strains (C57BL/6J and C57BL/10J). C57BL/10 substrains are in shades of red; C57BL/6 substrains are in shades of blue. *p-value < 0.05, **p-value < 0.01, assessed by ANOVA with Fisher's Least Significant Difference *post hoc* correction. Bars represent median phenotypes per substrain.

For total distance swum (Figure 5A), substrain effects were observed within the B10 branch but not within the B6 branch. Among B10 mice, C57BL/10J and C57BL/10ScSnJ mice swam shorter distances compared to C57BL/10SnJ and C57BL/10ScCr mice, and C57BL/10J also swam less than C57BL/10ScNHsd. There were no significant differences between the commonly used C57BL/6J and C57BL/10J strains (Table 3).

For swimming velocity (Figure 5B) substrain effects were not observed within the B10 or the B6 branch. There was no significant differences between the commonly used C57BL/6J and C57BL/10J strains (Table 3).

For the duration of “highly mobile” swimming (Figure 5C), which reflects periods of vigorous activity and is interpreted as an indicator of active coping, a significant effect of substrain was found within the B6 branch but not the B10 branch. C57BL/6ByJ and C57BL/6JBomTac mice spent more time swimming rapidly than C57BL/6NHsd and C57BL/6NJ. No significant differences were detected between the commonly used C57BL/6J and C57BL/10J (Table 3).

ANOVA results for all substrain-by-phenotype comparisons are reported in Table 2. *Post-hoc* results are reported in Supplementary Table 1.

## Discussion

In this study, we tested female mice from eight B6 and five B10 substrains of the C57BL lineage in a battery of behavioral tests to explore substrain-specific differences in behavior. Phenotypic differences that we observed are primarily attributable to genetic variation that distinguishes these substrains, including spontaneous mutations, genetic drift, and introgressed haplotypes due to breeding errors (Mortazavi et al., 2022).

Behavioral differences between selected B6 and B10 substrains have been demonstrated previously (Schimanski and Nguyen, 2005; Beery et al., 2018). For B6 substrains, behavioral differences have been shown in several previous works (Clapcote and Roder, 2004; Bothe et al., 2005; Bryant et al., 2008; Mulligan et al., 2008; Ashworth et al., 2015; Åhlgren and Voikar, 2019; Mekada and Yoshiki, 2021). The importance of substrain selection in C57BL/6 behavioral research has been highlighted by (Kiselycznyk and Holmes 2011), who noted robust substrain differences in locomotor activity, anxiety-related behavior, and prepulse inhibition of startle. However, there were no studies, to our knowledge, on behavioral differences in B10 substrains, and no studies comparing multiple B6 and B10 substrains in one set of tests. In addition, many previous works focused on male animals, while our work focused on female mice. The behavioral tests in the battery assess phenotypes that are widely used in animal models of multiple psychiatric disorders. They include behavioral responses to a novel environment, locomotor response to cocaine administration, response to fear stimulus, sensorimotor gating, and depression-like behaviors. Our work point out the importance of careful consideration of substrain selection depending on the research question and the behavioral test used to address that question.

Locomotor testing results demonstrated that both locomotor activity and exploratory behavior are shaped by substrain-specific genetic backgrounds, with pronounced differences observed within both B6 and B10 lineages. Importantly, C57BL/6J mice demonstrated a stronger exploratory behavior than C57BL/10J strain.

Acute cocaine exposure revealed notable substrain-specific differences in locomotor activity and exploratory behavior within the B6 lineage. The B10 substrains displayed more uniform responses, with no significant overall substrain effect. When examining anxiety-related behavior through changes in the novelty ratio, significant variability was observed among B6 substrains, while B10 substrains demonstrate largely consistent responses.

Fear conditioning (FC) is a classic Pavlovian learning paradigm in which an aversive unconditioned stimulus (US) is paired with a previously neutral conditioned stimulus (CS) and recall of the fearful memory is measured by observation of freezing behavior, a species-specific response to fear. The results of the FC test demonstrated lineage- and substrain-specific differences in both contextual and cued fear memory, with C57BL/10J mice consistently exhibiting stronger fear responses than other B10 substrains and the commonly used C57BL/6J.

Prepulse inhibition (PPI) was assessed to evaluate sensorimotor gating, a fundamental neurological process by which the brain filters sensory input to prevent cognitive overload. Deficits in PPI are linked to neuropsychiatric disorders such as schizophrenia and autism. We found substrain- and lineage-specific differences in both PPI and startle amplitude. C57BL/10J mice consistently exhibited lower PPI compared to other B10 substrains across all prepulse intensities.

FST results reveal that both B6 and B10 substrains exhibit some variability in their responses. The most pronounced differences found within the C57BL/10 lineage for distance, and within the C57BL/6 lineage for high-mobility swimming durations.

Taken together, these results demonstrate that substrain-specific genetic variation shapes behavioral phenotypes across multiple behavioral domains. The influence of genetic background across this diverse battery of assays underscores the importance of careful substrain selection and transparent reporting in preclinical studies that model psychiatric and substance use disorders.

Our findings are broadly consistent with previously reported behavioral differences among C57BL/6 substrains. We extended these observations to female mice and provided the first systematic behavioral comparison of B10 substrains. In the open field test (Matsuo et al., 2010) reported that C57BL/6J mice traveled the longest distance compared to C57BL/6N and C57BL/6C substrains, (Ashworth et al., 2015) similarly found that C57BL/6J male mice were significantly more active than C57BL/6N males, and (Bothe et al., 2005) found that C57BL/6J female mice have higher locomotor activity than C57BL/6Ntac female mice. Our results confirm this pattern in female mice, with C57BL/6J and C57BL/6JBomTac exhibiting the highest locomotor activity among B6 substrains, and C57BL/6J have higher total distance traveled that C57BL/6NTac. For fear conditioning (Bryant et al. 2008) reported that male C57BL/6J mice demonstrated a reduced level of conditional fear compared to C57BL/6N substrains. Our data similarly shows that C57BL/6J exhibited lower contextual and cued freezing than C57BL/10J, and that C57BL/6NCrl (an N-derived substrain) displayed higher pre-training freezing than C57BL/6J among B6 substrains. Similar to our findings, no difference was found between C57BL/6J and C57BL/6NTac in contextual and cued fear conditioning (Bothe et al., 2005). Regarding prepulse inhibition (Matsuo et al. 2010) found that C57BL/6N showed the highest prepulse inhibition among the substrains tested. Consistent with this, we observed that N-derived substrains C57BL/6NTac and C57BL/6NCrl exhibited the highest PPI among B6 substrains, while C57BL/6JBomTac showed the lowest. In the forced swim test (Matsuo et al. 2010) reported that C57BL/6J showed less immobility than C57BL/6N and C57BL/6C. While we did not observe significant substrain effects for distance or velocity among B6 substrains, we did find differences in high-mobility swimming measures.

Although phenotypic differences among B6 substrains have been reported previously, identifying the genetic variants that cause these differences remains challenging. One recent approach, called Reduced Complexity Cross (RCC) (Bryant et al., 2018; Babbs et al., 2019), involves mating closely related substrains that differ in a phenotype of interest, followed by crossing of the resulting F1 progeny to generate a recombinant F2 population. Crosses among closely related substrains segregate much fewer genetic variants than crosses between more divergent strains, thus making identification of causal alleles greatly simplified. A traditional F2 cross between divergent strains may contain thousands of candidate variants within a mapped genomic region, but an RCC narrows this to a much smaller number, permitting rapid identification of quantitative trait variants. This approach can be facilitated by use of inexpensive MiniMUGA microarrays that were designed for RCC mapping studies, which include polymorphic markers for many common substrains (Sigmon et al., 2020) or low-coverage whole genome approach (Polesskaya et al., 2025). Early genetic analysis based on limited number of SNPs detected minimal differences between B6 substrains (Tsang et al., 2005; Zurita et al., 2011). Now, with whole-genome sequence data available for C57BL/6 and C57BL/10 substrains (Mortazavi et al., 2022) and with inexpensive dense coverage genotyping method (Polesskaya et al., 2025), it is possible to map variants that give rise to various behavioral differences. Our study provides phenotypic data for the first step in this process: identifying substrain pairs with heritable trait differences suitable for RCC analysis, for example, the pronounced differences we observed between C57BL/6J and C57BL/6NTac in cocaine-induced locomotion, or between C57BL/10J and C57BL/10SnJ in fear conditioning.

This study is not without limitations.

First, we tested only female mice. Although we address an important gap in the literature where the majority of previous substrain comparison studies have focused exclusively on males, it also means that our findings may not fully generalize to male mice. Previous work has demonstrated that behavioral differences between B6 substrains can manifest differently in males and females (Ashworth et al., 2015). Future studies should examine whether the substrain differences we observed in females are also present in males, and whether any sex-by-substrain interactions exist for these behavioral phenotypes.

The second limitation is related to the nature of genetic variation between the substrains. Mortazavi et al. (2022) demonstrated that the majority of genetic differences between B6 and B10 lineages are not due to gradual accumulation of spontaneous mutations or genetic drift, but rather due to introgressed haplotypes resulting from historical breeding errors. These introgressed regions contain structural variants, protein-truncating SNPs, and frameshifting indels that may contribute to the phenotypic differences we observed. These large introgressed segments will likely emerge as major effect loci in any RCC analysis comparing B6 and B10 substrains. This limitation points out the importance of considering genetic architecture when designing RCC experiments, because genetic differences between closely related strains can arise through multiple mechanisms other than simple mutation accumulation.

The third limitation is that the estrous cycle was not monitored. Ovarian hormones fluctuate across the 4-day estrous cycle and are known to influence several of the behavioral phenotypes, potentially introducing hormonal state as an uncontrolled source of within-substrain variability. With respect to fear conditioning, estradiol has been shown to enhance contextual fear memory in female mice, and the phase of the estrous cycle prior to extinction training influences extinction recall (Milad et al., 2009). With respect to cocaine-induced locomotion, behavioral responses to psychostimulants reported to be affected by estrogen and being increased during proestrus and estrus phases (Sell et al., 2000; Van Swearingen et al., 2013). Anxiety-related behaviors are also modulated by estrous cycle modulation (Ryherd et al., 2023). The literature on effect of estrous cycle on prepulse inhibition is divided, with some studies reporting no significant effect (Charitidi et al., 2012) others report significant hormonal influence for this test (Plappert et al., 2005). In this study all substrains were tested under the same conditions without cycle synchronization, and mice were distributed across multiple testing cohorts. This design makes systematical bias due to hormonal state unlikely.

Fourth, although we bred all mice in-house and statistically controlled for testing covariates to minimize environmental confounds, we cannot fully exclude contributions from maternal effects, or litter effects, which were not controlled in this study. Different dams may exhibit variation in nursing behavior that could influence offspring phenotypes. Similarly, mice from the same litter share prenatal and early postnatal environments, potentially introducing non-genetic sources of phenotypic covariance. While the differences that we observed across multiple litters and cages are most parsimoniously explained by genetic variation, these potential confounds should be considered.

## Conclusion

These findings underscore the importance of considering genetic variations, even among closely related substrains, in experimental design and interpretation. We demonstrated that genetic divergence can significantly alter the number of phenotypes, including locomotor and anxiety-related behaviors, fear learning and memory, sensorimotor gating, and depression-like phenotypes. The accumulation of genetic differences over time can lead to significant phenotypic variability, which may impact the reproducibility and validity of research findings.

## Data Availability

The raw data supporting the conclusions of this article will be made available by the authors, without undue reservation.
